# How tapping works: physiological and psychological mechanisms in energy psychology

**DOI:** 10.3389/fpsyg.2025.1660375

**Published:** 2025-11-18

**Authors:** David Feinstein

**Affiliations:** Independent Practitioner, Ashland, OR, United States

**Keywords:** energy psychology, emotional freedom techniques (EFT), thought field therapy (TFT), acupoint tapping, physiological mechanisms, clinical efficacy, biomarkers, memory reconsolidation

## Abstract

Energy psychology protocols that incorporate tapping on acupuncture points—with Emotional Freedom Techniques (EFT) being the field’s most widely studied and used format—are contributing to an integration of psychological and somatic approaches in the treatment of conditions including PTSD, anxiety, and depression. Once dismissed as fringe or implausible, tapping therapies are now at the center of a scientific reckoning as mounting clinical and physiological evidence challenges longstanding skepticism. This review synthesizes over two decades of research—including more than 200 clinical trials, meta-analyses, biomarker investigations, and dismantling studies—to provide a comprehensive assessment of both the efficacy and mechanisms underlying acupoint tapping. The clinical trials have consistently led to positive outcomes, often with unusual speed and strong durability. Neurobiological research reveals that tapping generates electrochemical signals which modulate limbic as well as executive brain regions. These interventions also produce measurable shifts in biomarkers—including reduced cortisol, enhanced immune function, and changes in gene expression—that correspond with clinical improvements. Dismantling studies show that acupoint stimulation is a vital, non-placebo component of the approach. In addition, theoretical and empirical advances suggest that tapping protocols efficiently facilitate memory reconsolidation, enabling the durable transformation of distressing memories and maladaptive mental models. By mapping a stepwise physiological cascade from tapping to symptom relief, this review bridges the gap between clinical observation and scientific explanation, clarifies priorities for future research, and supports the integration of energy psychology into evidence-based mental health care.

## Graphical abstract

**Figure:**
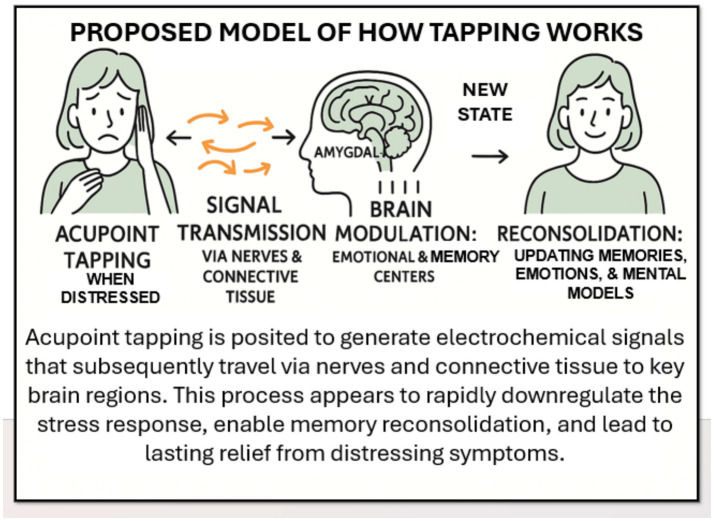

## 1 Introduction

Energy psychology has attracted more than its share of controversy. Prominent psychologists have, in top journals, referred to it as “pseudoscience” (Tolin et al., 2025) and warned that it is unethical for mental health professionals to practice it (Boness et al., 2023). The New York Times belittled it for looking “a little goofy” while summarily dismissing the entire body of research supporting its efficacy (Canon, 2025). When more than 11,000 practitioners and supporters signed a petition protesting Wikipedia’s disparaging coverage of the approach, founder Jimmy Wales publicly characterized the signers as “lunatic charlatans” (Wales, 2014), highlighting the intensity of opposition the field has faced. Yet despite such professional criticism and public derision, thousands of psychotherapists have found tapping to be unusually rapid and effective in relieving symptoms of stress, anxiety, depression, and PTSD.

The typical reaction to first experiencing or even just witnessing a tapping session is one of intrigued surprise. A client describes a personal difficulty or traumatic experience while rhythmically tapping their fingertips on points around the face and torso. To an outside observer, it can look like an odd blend of talk therapy and a children’s game—hardly what one expects from a serious clinical intervention. Yet, often to the observer’s surprise, the client reports feeling significantly better, sometimes within minutes. Even experienced mental health professionals who successfully use the method can find themselves wondering how such a simple, and at first glance almost counter-intuitive technique, might produce these effects. This paradox—impassioned doubt contrasted with consistently positive clinical outcomes—raises intriguing questions about what energy psychology is and how it works.

### 1.1 Definition

Energy psychology refers to a family of therapeutic approaches that generally integrate cognitive and somatic techniques, often involving the stimulation of electrochemically sensitive areas of the skin. The term “energy” in this context has both historical and contemporary meanings within the field’s development. When formulated in the 1980s, energy psychology drew from ancient Eastern healing systems which conceptualize health as involving the flow of subtle energies, such as qi in Chinese medicine or prana in Ayurveda. These notions of subtle energy, while foundational to the development of the field, remain controversial and are not readily measured by current scientific instruments, which has led to skepticism among many mental health professionals. Meanwhile, contemporary science explains “energy” in terms of established physiological and electromagnetic processes, such as measurable electrochemical signals and neural activity. Thus, while the origins of energy psychology were rooted in conceptualizations of subtle energies, current scientific models focus on mechanisms grounded in conventional biophysics and neurophysiology. Both perspectives are part of the field’s evolution (Feinstein, 2025; Leskowitz, 2024). This paper emphasizes empirically measurable mechanisms while acknowledging the broader conceptual heritage.

The vast majority of energy psychology research has focused on Emotional Freedom Techniques (EFT), the field’s most widely utilized format, and its predecessor, Thought Field Therapy (TFT). Both approaches employ the manual stimulation of acupuncture points (acupoints) by tapping on them while the client mentally activates a specific problem, memory, bodily sensation, or therapeutic goal. Tapping on specified areas of the skin generates electrochemical signals that initiate a cascade of physiological processes. During an acupoint tapping treatment, preliminary research suggests that these signals downregulate elevated limbic activity and update the mental models associated with psychological symptoms and challenges (Di Rienzo et al., 2020; Stapleton et al., 2019; Stapleton et al., 2022).

### 1.2 Structure of the paper

This paper reviews more than two decades of empirical findings and theoretical models that have investigated the physiological mechanisms underlying the favorable outcomes consistently described in clinical trials of energy psychology treatments. It surveys existing empirical evidence and proposes research directions for advancing this theoretical framework in four areas: (a) scientific evidence supporting efficacy, (b) the question of whether tapping on acupoints is an essential ingredient for positive outcomes, (c) biological markers observed after tapping treatments, and (d) the sequence of neurological and physiological processes that occur in a typical session, from tapping on the skin to clinical outcomes.

## 2 Efficacy

Before delving into the question of how tapping protocols work comes the question of *whether* they work. A closely related approach, acupressure, provides context for evaluating energy psychology treatments. Efficacy evidence for acupressure in treating mental health conditions is extensive. For instance, a recent systematic review and meta-analysis identified 14 randomized controlled trials (RCTs) with 1,439 participants, concluding that compared to standard care, acupressure treatments significantly reduced symptoms of depression and anxiety across diverse populations and clinical conditions (Lin et al., 2022).

Energy psychology protocols such as EFT and TFT stimulate some of the same points used in acupressure. These points may be activated by pressure, gentle touch, or rhythmic tapping (called “percussion” in the acupressure literature). Energy psychology protocols add distinct psychological components such as psychological exposure and cognitive restructuring, distinguishing them from traditional acupressure.

A database maintained by the Association for Comprehensive Energy Psychology (ACEP) contains more than 200 clinical trials investigating EFT or TFT published in peer-reviewed English-language journals, including 97 RCTs, plus an additional one hundred-plus clinical trials in other languages (Association for Comprehensive Energy Psychology, 2025). More than 99 percent of these 300 studies show statistically significant improvements on at least one targeted symptom or related issue. While this high success rate may reflect the influence of publication bias—where studies with null or negative results are less likely to be published—even when standard statistical corrections for publication bias were applied, the effects observed for acupoint tapping interventions remained robust (Seok and Kim, 2024). The 300 clinical trials, along with 11 meta-analyses and 11 additional systematic reviews cataloged by ACEP, provide substantial support for the efficacy of acupoint tapping protocols.

In addition to traditional meta-analyses that evaluate a single intervention against controls, comparative and network meta-analyses have emerged with the appearance of more comprehensive tools for assessing the relative efficacy of multiple therapies for the same condition. For example, Brown et al. (2017) and Mavranezouli et al. (2020) used this analytical approach to directly compare the outcomes of diverse interventions for trauma-based disorders. Both analyses found that tapping-based protocols performed not only as well as the established evidence-based treatments in the study, they were among the most effective of the comparative conditions, with particularly strong durability on follow-up. These comparative findings provide important context as the following discussion turns to the methodological rigor of the efficacy research.

### 2.1 The Tolin criteria

Comparing how this evidence base aligns with the stringent “Tolin criteria” for empirically supported treatments (Tolin et al., 2015) draws upon a rigorous standard for assessing the scientific credibility and clinical value of acupoint tapping protocols. The “Tolin criteria,” used by the American Psychological Association for evaluating psychological treatments, were developed to update and strengthen the process for identifying empirically supported treatments in psychology. Noting the dramatic increase in the number of published clinical trials, Tolin et al. created a system for considering the entire body of evidence when evaluating a treatment’s efficacy, emphasizing meta-analyses and other systematic reviews. They outlined detailed procedures for locating, screening, and assessing the quality of these multi-study investigations.

Tolin et al. also provided standards for evaluating the quality of the individual studies being assessed. As a group, the clinical trials investigating tapping protocols fulfill many of the Tolin criteria for clinical trials, particularly in the use of manualized protocols, standardized symptom measures, well-defined clinical populations, and a growing body of research demonstrating efficacy across multiple conditions. These studies increasingly include head-to-head comparisons with established therapies, utilize blinded evaluations, report long-term follow-up data, and examine interventions across diverse populations. In recent years, many studies have been conducted by independent investigators and parallel the conclusions of earlier studies conducted by proponents, addressing concerns about researcher allegiance. While many of the clinical trials cataloged by ACEP are consistent with the Tolin criteria’s emphasis on methodological rigor and generalizability, the Tolin framework, again, looks to reviews that critically appraise the quality of groups of studies investigating the same condition.

The 11 meta-analyses and 11 systematic reviews in the ACEP database demonstrate the field’s ongoing efforts to synthesize and evaluate the current body of evidence. Although earlier reviews did not fully conform to the stringent methodological requirements set forth in the Tolin criteria (Boness et al., 2023), recent analyses have applied more rigorous standards. Among these are meta-analyses concentrating on PTSD (Chen et al., 2025; Stapleton et al., 2023) and depression (Seok and Kim, 2024). These analyses fulfilled the Tolin criteria by employing systematic and transparent review methods, including only RCTs, finding large effect sizes, utilizing established tools to assess study quality, and consistently demonstrating benefits across diverse populations.

Among the most notable weaknesses shared by these meta-analyses are the reliance on self-report outcomes, risk of bias in the inclusion of studies, reliance on wait-list rather than active control conditions, and limited statistical power due to relatively small numbers of eligible studies. Despite these limitations and the need for more stringent systematic reviews, the existing meta-analyses demonstrate a consistent pattern of benefit across diverse populations and conditions. Moreover, while meta-analyses provide valuable synthesis, they are not the sole means for evaluating an evidence base.

### 2.2 The Cochrane handbook principle for converging evidence across studies

By placing primary emphasis on systematic reviews and meta-analyses, Tolin et al. acknowledge that their criteria may exclude effective interventions from consideration, “regardless of the quality of existing primary studies” (Tolin et al., 2015, p. 325). In contrast, the Cochrane Collaboration’s *Handbook for Systematic Reviews of Interventions*—the gold standard for systematic review methodology—provides another route for evaluating promising treatments that have not yet been assessed in high quality systematic reviews or meta-analyses. The *Handbook* emphasizes the evidentiary value of multiple studies yielding similar results even without systematic reviews: “The strength of evidence may be increased when several studies, each with some limitations, provide consistent findings in the same direction” (Higgins et al., 2019, p. 443). When a large number of smaller or less rigorous studies, conducted by different teams and using varied methods and data sources, independently produce similar outcomes in terms of benefit, harm, or no effect, this convergence becomes a compelling indicator of reliability. The ACEP database, with more than 200 English-language peer-reviewed clinical studies—and another hundred in other languages—demonstrates consistently strong effectiveness across diverse populations, research teams, and methodologies for a range of conditions. This broad, stable, and replicable pattern of positive outcomes in hundreds of studies, not just “several,” greatly reinforces the credibility and significance of the accumulated research on tapping therapies.

### 2.3 From efficacy to clinical implications: emerging research questions

As the efficacy base for acupoint tapping protocols matures, “process” (vs. “outcome”) research that has direct clinical implications takes on increased significance, shifting the focus toward questions that will inform best practices in real-world settings (Westra and Di Bartolomeo, 2024). For example, understanding what factors indicate whether individual or group treatments will be more effective for a particular client or condition can guide treatment planning. Determining when a basic tapping protocol is sufficient and when integration with other approaches is warranted helps clinicians make more informed choices. Clinical trials that include pre- and post-treatment measures of client characteristics and then correlate these with outcomes can illuminate which demographic, diagnostic, or personality groups are the most suitable candidates for tapping treatments. Addressing these questions will support the development of more personalized, flexible, and effective applications of tapping in mental health care.

## 3 Mechanisms

### 3.1 Proposed psychological and neurobiological mechanisms

This section examines proposed psychological and neurobiological mechanisms, drawing on dismantling studies, biomarkers, and neuroimaging research to explore how acupoint tapping may produce its reported outcomes. Understanding the mechanisms that might account for the rapid and lasting effects—reported in most studies that tracked speed and durability—requires investigation in three interrelated domains: (a) determining whether acupoint stimulation is an active treatment component, (b) identifying biological markers that shift following tapping interventions, and (c) mapping the sequence of neurological and physiological processes engaged during a typical session. The remainder of this paper synthesizes the empirical evidence for each domain, highlighting current knowledge and identifying priorities for future research.

### 3.2 The active ingredients question

The first issue in examining the proposed mechanisms of the approach is whether acupoint stimulation is an essential or even an active component in treatment outcomes. Such questions are most decisively addressed through dismantling studies—systematic investigations that compare a full protocol to versions with specific components removed or altered, thereby isolating their unique contributions. For example, outcomes after the complete EFT protocol can be compared with outcomes after removing acupoint tapping, replacing it with tapping on “sham” (non-acupuncture) points, or substituting another intervention such as diaphragmatic breathing or mindful attunement.

#### 3.2.1 Dismantling studies

A review of existing dismantling studies found that outcomes were superior when acupoint tapping was included (Feinstein, 2021), indicating that tapping is an active ingredient rather than a placebo or nonspecific factor. The earliest of these showed that participants with specific phobias who tapped on prescribed acupoints experienced significantly greater reductions in fear than those who used diaphragmatic breathing instead (Wells et al., 2003). Church and Nelms (2016) reported that, for frozen shoulder, acupoint tapping led to significantly greater improvements in pain, range of motion, and psychological symptoms than a similar protocol that substituted tapping with diaphragmatic breathing. In a partial replication of Wells et al., Fox (2013) found that students receiving EFT with acupoint tapping experienced significantly greater reductions in stress than those assigned to a mindfulness-based control condition, indicating that tapping contributed unique benefits beyond attentional focus alone. Because procedures such as diaphragmatic breathing or mindfulness differ substantially from tapping on the skin, however, they also in advertently alter other therapeutic components of the protocol.

The closest approximation for isolating the effects of acupoint stimulation is provided by studies that substitute tapping on the prescribed acupoints with tapping on “sham points,” non-acupoint locations. Three dismantling studies used sham-point controls, providing the closest methodological comparison for isolating the specific contribution of acupoint stimulation. In an investigation of teachers at risk for burnout, Reynolds (2015) found that EFT with acupoint tapping produced significantly greater reductions in emotional exhaustion and depersonalization, along with greater increases in personal accomplishment, over sham tapping. Working with veterans, Rogers and Sears (2015) reported that acupoint tapping resulted in significantly greater improvements in PTSD symptoms and overall functioning than sham-point tapping. Among postmenopausal women with depression, Mehdipour et al. (2021) found that EFT with acupoint tapping produced significantly greater symptom reductions than sham-point tapping.

Taken together, these three sham-point studies demonstrated stronger outcomes when prescribed acupoints were used, though limitations found in one or more of the studies included modest sample sizes, potential researcher allegiance, brief interventions, weaknesses in randomization, and non-identical instructions to the acupoint vs. sham groups.

#### 3.2.2 Tapping Without the Words

In addition to the evidence these dismantling studies provide, the conclusion that acupoint stimulation is an essential ingredient is further reinforced by a large-scale investigation involving 1,722 participants who received a single tapping session with only minimal conversation and none of the language prescribed in standard EFT protocols (Hamne et al., 2023). That study yielded highly significant reductions in self-reported anxiety and stress (*p* < 0.001), indicating that tapping alone can produce robust outcomes and highlighting the importance of further research into the mechanisms involved.

#### 3.2.3 Further investigation into acupoint tapping as an active ingredient

While dismantling studies to date generally support the view that acupoint tapping plays an active role in EFT outcomes, important questions remain about the precise therapeutic ingredients at play. What are the relevant contributions of tapping, exposure, and cognitive restructuring? Further dismantling studies that investigate this question and address the methodological critiques—such as ensuring adequately powered sample sizes, minimizing investigator allegiance, and carefully justifying outcome measures— would more firmly establish the place of acupoint tapping in producing EFT’s observed benefits.

### 3.3 Changes in biological markers following tapping treatments

Building on the discussion of efficacy and active ingredients, a third line of inquiry concerns shifts in biological markers following tapping treatments. Such markers—objective indicators like hormone levels, immune function, and heart rate variability—offer a window into the physiological shifts associated with EFT protocols. While these findings underscore the body’s positive response to tapping, they primarily serve as signs of improvement rather than explanations of underlying mechanisms. In other words, these biomarkers reveal important physiological shifts that accompany clinical benefits, but they do not, by themselves, explain how acupoint tapping produces these effects—a topic addressed in greater depth in the final sections of this paper.

#### 3.3.1 Biomarker research

A growing body of research has documented significant changes in biological markers following EFT and related acupoint tapping interventions. These physiological shifts often parallel improvements in physical and psychological symptoms. Because such markers provide objective evidence of physiological change, they offer corroboration of the clinical benefits subjectively reported by patients and observed by clinicians.

##### 3.3.1.1 Cortisol

Among biomarkers, cortisol has received the most attention. In a landmark study involving 83 participants, Church et al. (2012) found significant reductions in salivary cortisol following a single EFT session, and these reductions were considerably greater than those observed with supportive counseling or no treatment controls. The EFT group showed a mean cortisol decrease of 24.4% compared to 14.2% for the counseling and 0.6% in no-treatment controls. This study was replicated by Stapleton et al. (2020) who found an even larger cortisol reduction (43%) in the EFT group, again substantially exceeding changes in the psychoeducation and no-treatment conditions. These results suggest that EFT is not only effective in reducing stress-related biochemistry but does so more robustly and more rapidly than standard talk-based interventions.

##### 3.3.1.2 Immune function

Statistically significant increases in immunoglobulin A (IgA) and other biochemical sources of immunity have been observed post-intervention, suggesting enhanced immune function following tapping protocols (Babamahmoodi et al., 2015; Bach et al., 2019).

##### 3.3.1.3 Heart rate variability

Improvements in heart rate variability, reflecting better autonomic regulation, have been reported following tapping treatments (Bach et al., 2019; Hidayat et al., 2021).

##### 3.3.1.4 Gene expression

Preliminary evidence suggests that EFT may beneficially influence gene expression related to immunity, stress, emotional regulation, synaptic function, and brain connectivity (Church et al., 2018; Maharaj, 2016).

##### 3.3.1.5 Other biomarkers

Clinically meaningful improvements in blood pressure, heart rate, and EEG patterns have also been observed, further illustrating the diverse physiological systems influenced by tapping interventions (Bach et al., 2019; Lambrou et al., 2003; Swingle et al., 2004).

#### 3.3.2 Directions for further biomarker research

While the appearance of these biological markers following tapping treatments is well-established, further research will deepen understanding of their mechanisms, clinical relevance, and the precise procedures that catalyze them.

Larger, well-controlled studies across diverse populations and clinical conditions would confirm existing findings on biomarker changes. Such studies would also enhance generalizability by identifying samples that are representative of specific clinical and demographic populations.

Consistent protocols for biomarker measurement (including timing, type of sample, and analytic methods) will enhance comparability and replicability across studies.

Further research could also reveal how changes in specific biological markers—such as cortisol, gene expression, and heart rate variability—relate to psychological outcomes and long-term health and mental health improvements.

Further research could clarify how observed biomarker shifts are connected to the underlying neurological and physiological processes engaged by acupoint tapping. Integrating biomarker findings with neuroimaging and other physiological measures would move the field beyond simply demonstrating that tapping produces biological changes and toward understanding the clinical significance of those changes, along with the specific pathways through which they lead to treatment gains.

### 3.4 The sequence of neurological and physiological processes in a typical session

This section turns to proposed sequences of neurological and other physiological processes that may occur during a typical tapping session. For most psychotherapies, and even for biological interventions such as antidepressant medication or electroconvulsive therapy, the precise sequences of neural and biochemical events leading to clinical improvements are not yet fully understood (Carey et al., 2021; Holmes et al., 2014; Insel, 2015). Despite decades of research into the mechanisms of a particular treatment, the medical field often relies on broad theoretical models rather than detailed step-by-step descriptions of how its interventions catalyze change at the physiological level.

In the face of uncertainty surrounding the mechanisms question, energy psychology has—like many psychotherapeutic approaches—developed speculative theoretical models to explain the actions of its interventions. This issue is particularly salient for acupoint tapping, which looks odd, is unfamiliar to most traditionally-trained psychotherapists, and whose relationship to psychological change is by no means obvious, if not counter-intuitive. The inability to present plausible, empirically-supported mechanisms has, in fact, been a significant impediment to the field’s broader acceptance (Mitchell and Chatzidamianos, 2020).

However, a growing body of research has begun to provide an evidence base for the evolving theoretical models about the actions of tapping. Studies have documented neurological and other physiological changes, lending empirical support for the proposed pathways by which acupoint stimulation may lead to clinical outcomes (Stapleton et al., 2019; Stapleton et al., 2022; Wittfoth et al., 2020).

The following five premises outline the sequential physiological and neurological events believed to underlie the effects of acupoint tapping. They are presented in the order in which the processes they describe unfold during a typical tapping session. For each premise, the existing empirical support is briefly reviewed, followed by a discussion of additional research that would further substantiate or clarify the proposed mechanism.

#### 3.4.1 Sequence premise 1: the stimulation of acupoints generates distinct electrochemical signals

Stimulating an acupoint produces electrochemical signals that are significantly stronger than those generated by tapping on non-acupoint areas due to the higher electrical conductivity and greater density of mechanosensory receptors found at acupoints compared to adjacent tissue (Ahn et al., 2008; Han, 2023). Mechanosensory receptors convert mechanical stimulation, in this case tapping, into electrical activity. This well-established process, known as mechanosensory transduction, instigates a cascade of neural signaling (Sala et al., 2024). These early-stage electrochemical events in tapping protocols are believed to initiate the physiological and psychological outcomes that have been consistently observed in clinical trials (Church et al., 2022).

Of the 361 primary acupuncture points identified by the World Health Organization (1991), the manual used in TFT research (Callahan and Callahan, 2001) typically specifies only 5 to 7 points during a round of tapping, with the specific points varying depending on the condition being treated (anxiety, panic, depression, trauma, anger, resentment, guilt, grief, etc.). A key distinction between TFT and EFT is that EFT employs a standardized, “one-size-fits-all” sequence of nine acupuncture points for all conditions (Church, 2018), rather than tailoring the points to specific issues as is done in TFT. This difference is promoted as an advantage for simplicity, ease of teaching, and research consistency, but it also represents a major departure from the individualized point selection characteristic of TFT as well as from classical Chinese medicine. While this approach offers clear advantages for ease of application and training, EFT practitioners often adapt the standardized sequences—modifying the number, selection, and order of points to meet a clients’ immediate needs. Empirical investigations of TFT and EFT show strong outcomes for each. Future research could, however, help determine whether the points prescribed for specific conditions in TFT are optimally matched for treating those conditions, whether the one-size-fits-all claim of EFT is substantiated, and, if so, what might constitute the ideal points and sequences for a simple universal protocol.

If this line of research proves instructive for improving outcomes, future investigation using advanced imaging might address foundational questions about the effects of stimulating specific points or sets of points. For instance, studies that isolated each TFT and EFT point could map the pathways from tapping on specific points to their neurological and psychological effects, facilitating comparisons and informing point selection for clinical protocols. Other questions such as the optimal durations for tapping on each point and the order of points used in a sequence could similarly be investigated. While existing protocols have been shown to be highly effective across a range of conditions, such research would strengthen understanding of the physiological underpinnings of energy psychology treatments and advance the refinement of clinical practice.

#### 3.4.2 Sequence premise 2: brain regions related to the memories, thoughts, and emotions the client brings to mind during the tapping are activated

During acupoint tapping sessions, clients focus on specific memories, thoughts, or emotions. This focus activates associated brain regions and engages corresponding neural circuits involved in emotional processing (Logothetis, 2008; Shin and Liberzon, 2010). This activation occurs independent of any acupoint stimulation but is an important step in engaging the brain’s emotional regulation systems, creating conditions for the neurological effects associated with tapping. It appears to prime the neural circuits for modulation by the subsequent physiological signals generated during tapping.

#### 3.4.3 Sequence premise 3: signals generated by tapping are transmitted to the brain via afferent nerves and connective tissue

When individuals focus on a problem or goal during tapping, recalling the issue activates neural circuits involved in processing related memories, emotions, and thoughts. Simultaneously, the mechanical stimulation from tapping the skin produces electrochemical signals through mechanotransduction, transmitted to the brain via afferent sensory nerves (Delmas et al., 2011; Rocha et al., 2022). These signals are also propagated through the body’s connective tissue network (Maurer et al., 2019), which appears to function as a bodywide mechanosensitive signaling system capable of transmitting mechanical and electrical disturbances over long distances (Ingber et al., 2006).

Connective tissue contains high concentrations of semiconductive collagen fibers that transmit electricity and are concentrated around acupoints (Ding et al., 2017). This dual system of neural and connective tissue transmission enables rapid propagation of signals from the skin to the brain, reaching the brain regions that have been activated by the client’s focused attention during the tapping. This convergence of physiological signaling with attentional activation suggests a plausible neurobiological pathway that may help explain the rapid and targeted psychological and physiological effects reported in many studies of acupoint tapping.

A longstanding source of controversy in energy psychology has been the concept of meridians, described in traditional acupuncture theory as “energy pathways” (Ahn et al., 2008). Critics have often dismissed meridians as lacking anatomical reality (Boness et al., 2023). However, the physiological mechanisms described here do not rely on meridian theory. Instead, the focus is on empirically supported biophysical pathways—afferent nerves and connective tissue networks—that can be measured and mapped with conventional scientific instruments. Nonetheless, advances in imaging and bioelectrical measurement (including electroconductivity, hydraulic conductance, and thermal and acoustic signal propagation) have revealed anatomical structures and electrical conductance patterns that correspond with traditional meridian diagrams (Nan et al., 2023; Yunshan et al., 2025). Current understanding increasingly situates meridian-like pathways within connective tissue matrices (Langevin and Yandow, 2002; Li et al., 2020). While debate remains, these findings challenge the notion that meridians are fictional, providing substantial scientific support for their anatomical basis.

Independent of any consideration of meridians, further imaging and electrophysiological research could clarify the precise pathways by which signals generated at acupoints are transmitted through both neural and connective tissue networks. Such studies could determine whether tapping on a prescribed EFT or TFT point produces distinct patterns of electrical activity or signal propagation and whether differences among points in their capacity to generate and transmit signals correlate with differences in clinical outcomes. Direct comparisons of neural versus connective tissue pathways could establish the relative contributions of each route in bringing about therapeutic effects. Additionally, elucidating how these physiological signals interact with attentional and cognitive processes would provide a more comprehensive understanding of the psychological and physiological effects that have been observed in acupoint tapping studies. Addressing these questions would deepen understanding of the physiological mechanisms underlying energy psychology protocols and inform the development of more precise and effective interventions.

#### 3.4.4 Sequence premise 4: signals generated by acupoint tapping upregulate or downregulate clinically relevant brain regions, promoting homeostasis, balance, and adaptive behavior

A 10-year research program at Harvard Medical School demonstrated that stimulating acupuncture points with needles can rapidly modulate both limbic and executive brain regions (Fang et al., 2009; Hui et al., 2009). The investigators found that certain acupoints produced almost instant deactivation of the limbic–paralimbic–neocortical system while others activated executive regions such as the dorsolateral prefrontal cortex, middle and superior frontal gyri, and anterior cingulate cortex. Some points simultaneously downregulated some brain regions while upregulating others. Because these findings are based on traditional needling of acupuncture points, their relevance to acupoint tapping, which involves less intense mechanical stimulation remains uncertain. Nonetheless, they provide a useful context for considering possible mechanisms of tapping. Neuroimaging studies of tapping itself are beginning to show how acupoint tapping influences brain activity in similar ways (Stapleton et al., 2019; Stapleton et al., 2022; Di Rienzo et al., 2020).

**Figure 1:**
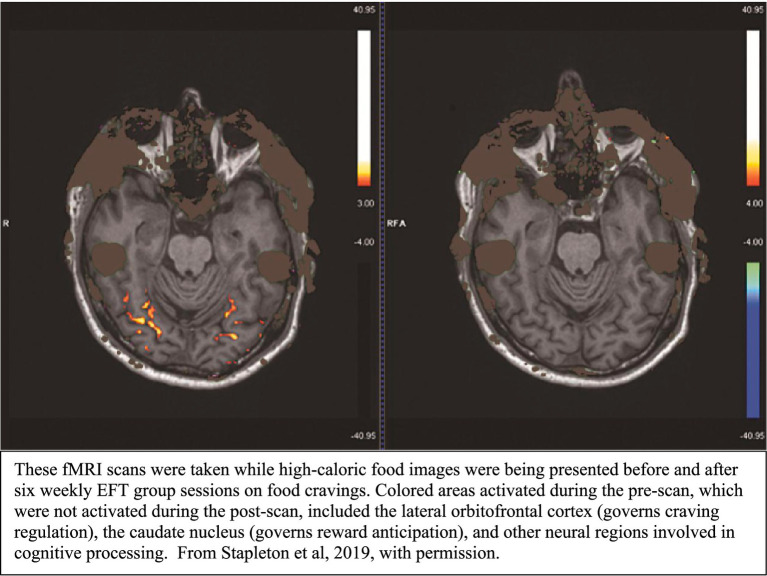
Pre- and post-treatment fMRI scans.

Once the signals generated by tapping reach the brain regions activated by the client’s recalled memory, problem, or goal, they initiate immediate neural modulation within those circuits. The imaging studies of acupoint tapping indicate that tapping can both downregulate areas linked to emotional distress and upregulate regions associated with cognitive control and adaptive behavior. For example, separate studies showed how EFT altered neural activity in individuals with food cravings (Stapleton et al., 2019) and chronic pain (Stapleton et al., 2022). Both studies reported that downregulation of these regions was associated with and appeared to lead to symptom improvement in participants (see Figure 1).

Extending this line of evidence, a pioneering case study by Di Rienzo et al. (2020) found that EFT tapping in the treatment of a phobia was associated not only with downregulation of limbic activity but also with upregulation of prefrontal regions involved in cognitive control and adaptive behavior. Alongside these neural effects, hormonal findings indicate that EFT modulates the hypothalamic–pituitary–adrenal (HPA) axis—the body’s central stress-response system—by reducing cortisol and restoring neuroendocrine balance (Church, 2013).

Overall, this early brain imaging and hormonal evidence suggests that acupoint tapping treatments modulate symptom-relevant biological systems in ways consistent with clinical observations of rapid emotional and behavioral change. Imaging studies offer the opportunity to systematically map how acupoint tapping influences the neural landscape associated with specific clinical conditions and to determine whether certain acupoints or tapping sequences are more effective than others for modulating these pathways. Such research would clarify whether neural changes induced by tapping are consistent across diagnoses, symptom profiles, or individual neurobiology.

An especially intriguing question is how the same acupoint can downregulate activity in some brain regions while upregulating others, as found in the Harvard research program and suggested in the Di Rienzo case study. Further investigation could also explore whether particular acupoints or sequences produce more pronounced effects for certain psychological conditions, and how individual neurobiological differences shape the direction and magnitude of these neural changes. Clarifying these issues would help determine whether the effects of acupoint tapping arise from the unique properties of individual points, the combined action of multiple points, or broader regulatory mechanisms.

#### 3.4.5 Sequence premise 5: acupoint tapping protocols facilitate transformative change in unprocessed distressing memories and maladaptive mental models

A robust body of research demonstrates that acupoint tapping protocols produce rapid and significant symptom relief across a range of clinical conditions (Association for Comprehensive Energy Psychology, 2025; Church et al., 2022). In studies that have included follow-up assessments, these improvements have not only been rapid but have also shown lasting effects over time. Deep-seated emotional learnings can be transformed through a neurological process known as memory reconsolidation (Ecker et al., 2012; Lane et al., 2015; Phelps and Hofmann, 2019).

In memory reconsolidation, activated memories or mental models become temporarily labile and can be updated or erased if a significant mismatch—known as a prediction error—contrasts what is expected from what is actually experienced. When clients recall previously distressing material while experiencing a marked reduction in emotional arousal due to the acupoint tapping, this pronounced sense of calm sharply contrasts with the anticipated distress. This discrepancy—the prediction error—signals the brain that the old emotional response is no longer valid, enabling the memory or mental model to be emotionally reconsolidated in a less distressing, more adaptive form that endures long-term. *Emotional* memory reconsolidation—where the factual details remain intact but the emotional response changes—differs from *declarative* memory reconsolidation, in which the factual content itself is modified (Pedreira, 2013). This distinction is particularly relevant to EFT protocols.

The emotional reconsolidation framework offers a strong theoretical foundation for understanding the neurological mechanisms behind tapping protocols. However, further empirical research could clarify how prediction errors arise and drive cognitive updating during acupoint tapping. For instance, studies that map the neurological precursors and real-time dynamics of prediction errors—such as changes in amygdala reactivity or prefrontal activation during tapping—could reveal how emotional reconsolidation contributes to clinical improvements. Research that tracks emotional and physiological responses during distressing memory recall, with and without tapping, would directly test the role of acupoint stimulation in generating prediction errors and supporting lasting reconsolidation. Comparative studies with other therapies could clarify the unique role of reconsolidation in tapping’s effectiveness, while longitudinal imaging studies would determine whether neural changes in memories or mental models persist and how they relate to the sustained clinical benefits that have been consistently reported.

An intriguing clinical observation is that therapeutic suggestions or self-directed affirmations appear to have amplified effects when combined with tapping. It is as if an affirmation such as “feeling more peaceful about what happened” is being “tapped” into the nervous system, somatically reinforcing the therapeutic impact of the words. Empirical investigation into this phenomenon could determine whether tapping enhances the integration of therapeutic suggestions within relevant neural circuits, thereby increasing the durability and depth of therapeutic change. If confirmed, this would represent a significant advance in understanding how somatic and cognitive elements can be synergistically combined to promote lasting psychological transformation.

### 3.5 Summary of mechanisms

Taken together, these findings suggest a neurophysiological framework in which acupoint tapping may act as a catalyst for a cascade of biological and psychological changes. While the precise mechanisms remain under investigation, one hypothesis is that mechanical stimulation at specific acupoints generates electrochemical signals in peripheral sensory nerves and in mechanosensitive connective tissue. These signals are thought to travel through established neural pathways and fascial networks to limbic and cortical regions involved in emotional regulation and threat processing, including the amygdala, hippocampus, and prefrontal cortex.

Such signals are thought to modulate limbic system activity, promoting a shift toward parasympathetic nervous system activation and lowering stress hormone levels. This neurophysiological cascade may support the reprocessing and updating of distressing memories and maladaptive mental models through mechanisms such as emotional reconsolidation. Dismantling studies suggest that acupoint stimulation is an essential ingredient in producing these effects, and a broad range of biomarker changes—including reductions in cortisol, improvements in immune function, and shifts in gene expression—have been reported. While the precise sequence and interactions of these processes require further empirical validation, emerging evidence suggests that tapping may trigger interconnected neural and physiological processes that contribute to symptom improvement.

## 4 Discussion

To date, research in energy psychology has necessarily focused on establishing efficacy and documenting biomarker changes, underlying a field’s need to demonstrate that its distinguishing methods work before fully investigating how they work. Now that an encouraging efficacy base has been established for acupoint tapping protocols, the field has continued to expand its investigation of effectiveness across new populations and conditions, even as it increasingly turns attention to understanding the physiological mechanisms that account for these outcomes. Emerging evidence suggests that these processes can be conceptualized along a sequential pathway:

1. Generation of electrochemical signals by acupoint tapping.
2. Activation of brain regions related to the memories, thoughts, and emotions the client brings to mind during the tapping.
3. The signals generated during tapping are believed to be transmitted to these attention-activated brain regions.
4. Neural modulation within these regions is initiated by the arrival of the transmitted signals.
5. Durable modification of unprocessed distressing memories and maladaptive mental models, purportedly through the reconsolidation process.

This stepwise framework not only organizes current findings but also guides future research priorities. Understanding the neurological and other physiological mechanisms also enables nuanced comparisons with other therapeutic approaches. For example, although exposure techniques are used in both energy psychology and cognitive behavior therapy (CBT), the distinct neurological processes involved in tapping protocols—such as the generation of signals that promote rapid modulation of limbic activity and facilitate memory reconsolidation—help clarify the differing actions and outcomes of these interventions.

### 4.1 Conventional exposure

Conventional CBT exposure protocols reduce fear and avoidance by systematically confronting distressing memories or stimuli, relying primarily on habituation and extinction processes (Foa and McNally, 1996; Craske et al., 2018). This approach often requires repeated, prolonged sessions to achieve meaningful symptom reduction, a process that can be emotionally taxing for clients and is associated with relatively high drop-out rates (Lewis et al., 2024). The underlying mechanism involves forming new inhibitory associations that compete with—but do not erase—the original fear memory (Foa and McNally, 1996; Craske et al., 2018). Consequently, under stress or in novel contexts, the original fear response can resurface through spontaneous reactivation or relapse (Lane et al., 2015).

### 4.2 Exposure with tapping

In contrast to CBT protocols for exposure, energy psychology combines psychological exposure with acupoint stimulation, engaging additional neurobiological processes. Tapping on specific acupoints during the recall of distressing memories sends electrochemical signals through peripheral nerves and connective tissue to brain regions involved in threat detection and emotional regulation, including the amygdala, hippocampus, and prefrontal cortex (Di Rienzo et al., 2020; Stapleton et al., 2019; Stapleton et al., 2022). This stimulation rapidly reduces limbic arousal and promotes a shift from sympathetic activation toward greater parasympathetic activity, as evidenced by decreased cortisol (Church et al., 2012; Stapleton et al., 2020) and increased heart rate variability (Bach et al., 2019). The resulting calming effect allows clients to remain emotionally regulated during exposure, reducing the risk of retraumatization and fostering a sense of safety.

Crucially, this integration of exposure and acupoint tapping facilitates emotional reconsolidation—the process by which memories are not simply suppressed but neurologically updated. When a distressing memory or related beliefs are reactivated in a safe, tapping-induced state of calmness, and a prediction error occurs (e.g., expected distress fails to materialize), the brain updates the emotional associations to the memory (Ecker et al., 2012). Meta-analytic evidence confirms that this mechanism produces more durable symptom relief and lower relapse risk than traditional exposure (Stapleton et al., 2023). In survivors of catastrophic events, acupoint tapping has been found to rapidly reduce acute distress and persistent symptoms—even in highly traumatized populations—with significant improvements frequently observed after just one or a few sessions (Feinstein, 2022; Hamne et al., 2023; Nemiro and Papworth, 2015).

### 4.3 Integrating tapping with other treatments

Understanding these mechanisms clarifies why exposure combined with acupoint tapping yields faster, less traumatizing, and longer-lasting outcomes than exposure alone (Stapleton et al., 2023). By directly targeting neurobiological pathways involved in emotional regulation and memory updating, these interventions offer a distinct therapeutic advantage—particularly for individuals who may find traditional exposure overwhelming or insufficiently effective (Church et al., 2018; Clond, 2016; Stapleton et al., 2023). These principles extend far beyond exposure. While EFT and TFT can be used as independent treatment modalities, when addressing serious disorders such as PTSD, chronic depression, or addictions, combining tapping with existing best practices holds considerable promise for optimizing clinical outcomes (Feinstein, 2023).

### 4.4 Process research

A recent advance within psychotherapy is the rise of process research—studies that move beyond outcome measurement to examine how therapists deliver interventions most effectively. Westra and Di Bartolomeo (2024) have proposed that the skills developed by process researchers—such as identifying critical therapist decision points and coding session dynamics—should also be incorporated into clinical training programs. By cultivating these skills, psychotherapists can better recognize which of their behaviors increase engagement, enthusiasm, or encouragement, and which may lead to disengagement, resistance, or ruptures in the therapeutic alliance.

Process research within energy psychology could examine the specific types of statements therapists introduce as the client taps and the choices that improve treatment outcomes. A preliminary study showed that wording accompanying tapping which was judged as moving the session forward served one of three basic functions: attuning, exploring, or directing (Feinstein, 2019). Process research can further examine the ways practitioners word their statements, when to encourage deeper emotional processing or pause for integration, how to recognize and respond to signs of client distress or potential overwhelm, how to tailor pacing and focus to individual needs, when to introduce cognitive reframing, and how to maintain therapeutic momentum. Each of these moment-to-moment decisions may influence the degree to which tapping protocols achieve therapeutic benefit. Future research could also clarify whether differences in point selection, sequence, or intensity of tapping contribute meaningfully to treatment outcomes, or whether the core mechanisms operate independently of such variations.

## 5 Conclusion

Energy psychology protocols that incorporate acupoint tapping, such as EFT and TFT, have advanced from the margins toward mainstream clinical practice, demonstrating rapid and durable benefits across a range of conditions. This review outlines a provisional framework for how tapping may work—from the generation of electrochemical signals to the modulation of brain regions involved in emotional processing, neurologically transforming of distressing memories and maladaptive patterns. By synthesizing recent clinical, neurobiological, and biomarker evidence, this review provides a plausible science-based framework for the mechanisms that may contribute to tapping’s effects. It also identifies priorities for future research to strengthen the field’s empirical foundation. While the precise mechanisms remain under investigation, a growing body of clinical and laboratory evidence provide a framework for understanding why energy psychology is making a meaningful contribution to evidence-based practice and mental health care.
